# Proliferation of Inhibitory Input to the Substantia Nigra in Experimental Parkinsonism

**DOI:** 10.3389/fncel.2019.00417

**Published:** 2019-09-13

**Authors:** Anna Faynveitz, Hagar Lavian, Avi Jacob, Alon Korngreen

**Affiliations:** ^1^The Mina and Everard Goodman Faculty of Life Sciences, Bar Ilan University, Ramat Gan, Israel; ^2^The Leslie and Susan Gonda Interdisciplinary Brain Research Center, Bar Ilan University, Ramat Gan, Israel

**Keywords:** substantia nigra, short-term depression, GABA, 6-OHDA, long-term plasticity, synaptic proliferation

## Abstract

The substantia nigra pars reticulata (SNr) is one of the output nuclei of the basal ganglia (BG) and plays a vital role in movement execution. Death of dopaminergic neurons in the neighboring nucleus, the substantia nigra pars compacta (SNc), leads to Parkinson's disease. The ensuing dopamine depletion affects all BG nuclei. However, the long-term effects of dopamine depletion on BG output are less characterized. In this *in vitro* study, we applied electrophysiological and immunohistochemical techniques to investigate the long-term effects of dopamine depletion on GABAergic transmission to the SNr. The findings showed a reduction in firing rate and regularity in SNr neurons after unilateral dopamine depletion with 6-OHDA, which we associate with homeostatic mechanisms. The strength of the GABAergic synapses between the globus pallidus (GP) and the SNr increased but not their short-term dynamics. Consistent with this observation, there was an increase in the frequency and amplitude of spontaneous inhibitory synaptic events to SNr neurons. Immunohistochemistry revealed an increase in the density of vGAT-labeled puncta in dopamine depleted animals. Overall, these results may suggest that synaptic proliferation can explain how dopamine depletion augments GABAergic transmission in the SNr.

## Introduction

The substantia nigra is one of the basal ganglia (BG) nuclei located in the ventral midbrain and is divided into the substantia nigra pars compacta (SNc) and the substantia nigra pars reticulata (SNr). The SNc contains neurons projecting to the striatum and modulate striatal activity by releasing dopamine (Freund et al., 1984), while the SNr projects primarily to the thalamus, and serving as one of the primary output nuclei of the BG (Albin et al., 1989). This output nucleus plays a critical role in motor activity, particularly of the eyes and head (Sakamoto and Hikosaka, 1989; Mink, 2003; Dybdal et al., 2013). The SNr is populated by GABAergic and dopaminergic neurons, which can be distinguished by their electrophysiological properties (Richards et al., 1997). Anatomically, SNr GABAergic neurons project to the thalamus, the superior colliculus, and the pontine reticular formation (Carpenter et al., 1976; Beckstead and Frankfurter, 1982; Beckstead, 1983). Functionally, SNr GABAergic neurons receive excitatory input from the subthalamic nucleus (STN) (Kita and Kitai, 1987) and inhibitory input from the striatum (STR) and the globus pallidus (GP) (Grofova, 1975).

Parkinson's disease (PD) is a degenerative movement disorder of the dopaminergic neurons in the SNc, which includes motor symptoms such as rigidity, bradykinesia, and tremor (Ehringer and Hornykiewicz, 1960). Apoptosis of dopaminergic neurons in the SNc affects all BG nuclei. Dopamine influences information integration by the SNr in various ways. SNc dopamine neurons increase the activity of the striatonigral pathway via the D1 receptor and decrease the activity of the striatopallidal pathway via D2 receptors (Gerfen et al., 1990). Overall, the two pathways reduce the activity of the SNr GABAergic neurons (Gerfen and Surmeier, 2011). Also, dopaminergic neurons extend their dendrites into the SNr (Björklund and Lindvall, 1975; Henny et al., 2012). Dopamine directly released from dendrites of the SNc dopaminergic neurons modulates the activity of GABAergic neurons (Geffen et al., 1976; Cheramy et al., 1981).

In *in vitro* experiments, blockage of D_1_ and D_5_ receptors was shown to reduce SNr activity and regularity by inactivation of the ultra-short SNc-SNr pathway (Zhou et al., 2009). Also, pharmacological manipulation of dopamine receptors changes inhibitory synaptic activity. Studies have shown that a D1 agonist enhanced the striatum-SNr (STR-SNr) IPSC but had no effect on the GP-SNr synapse. Conversely, the D2 agonist did not affect STR-SNr IPSC but depressed the GP-SNr synapse (Aceves et al., 2011). Since dopamine depletion affects the entire BG (Azdad et al., 2009; Ketzef et al., 2017), the influence of neuronal death on the intrinsic and synaptic properties of GABAergic neurons have been investigated in dopamine depleted animals. The findings indicate an increment in bursts in *in vivo* experiments (Wichmann et al., 1999; Lee et al., 2001; Wang et al., 2010a). Other studies have reported an increment, a decrement or no change in spontaneous firing (Sanderson et al., 1986; Murer et al., 1997; Rohlfs et al., 1997; Díaz et al., 2003; Breit et al., 2008; Wang et al., 2010b). Recent *in vitro* work indicated a decrease in firing rate and an increase in irregularity and bursting behavior of these neurons (Cáceres-Chávez et al., 2018).

The current *in vitro* study was designed to better understand the influence of dopamine depletion on homeostatic and synaptic plasticity of GABAergic neurons in the SNr. We found changes in the firing properties of these neurons in 6-OHDA adult rats during blockage of the whole network. In addition, we investigated the synaptic alterations of dopamine depletion in the GP-SNr synapse and changes in inhibitory input probabilities. We also characterized anatomical changes in the number of inhibitory puncta in the SNr as a possible mechanism to account for the increase in release after dopamine depletion.

## Materials and Methods

### Unilateral Dopamine Depletion

Surgeries were carried out on 180–220 g (8–12 week old) Wistar rats. We performed all experiments according to the guidelines of the Bar-Ilan University Animal Welfare Committee. The procedures were approved by the National Committee for Experiments on Laboratory Animals at the Israeli Ministry of Health. The rats were initially anesthetized with isoflurane and then maintained under anesthesia with ketamine (1 ml/kg) and xylazine (0.5 ml/kg). Twenty-five minutes before 6-OHDA injection, the animals received an injection of the monoamine oxidase inhibitor pargyline (50 mg/kg, IP) which augments the toxic effect of 6-OHDA on dopaminergic cells by preventing its degradation by endogenous enzymes. We placed the animals in a stereotaxic instrument and made a small craniotomy. 6-OHDA containing 0.01% w/v ascorbic acid (3 mg/ml) was dissolved immediately before use. A total amount of 4.5 μl of neurotoxin was injected at a rate of 0.5 μl/min into the region adjacent to the medial SN at coordinates indicated in the Paxinos and Watson rat brain atlas (4.5 mm posterior to the bregma, 1.6 mm lateral to the midline, and 7.9 mm ventral to the dura). A behavioral test, conducted 14 days after the surgery by injecting apomorphine (0.05 mg/kg), estimated the severity of the lesion. Following apomorphine injection, dopamine depleted animals turned 7.1 ± 2.4 turns/min (*n* = 33) in the direction contralateral to the damaged hemisphere while only 0.1 ± 0.08 turns/min in the ipsilateral direction. In this study we only used rats that rotated contralateral from the injected side by at least 5 turns/min.

### *In vitro* Slice Preparation

Brain slices were obtained from 8 to 12 week old Wistar rats, as previously described (Bugaysen et al., 2010; Ting et al., 2014; Gorodetski et al., 2018). We lightly anesthetized rats with isoflurane followed with a deeper anesthesia by injection of ketamine (1 ml/kg) and xylazine (0.5 ml/kg). Transcardial perfusion was performed with NMDG artificial CSF (ACSF) containing the following (in mM): 92 NMDG, 2.5 KCl, 1.25 NaH_2_PO_4_, 30 NaHCO_3_, 20 HEPES, 25 glucose, 2 thiourea, 5 Na-ascorbate, 3 Na-pyruvate, 0.5 CaCl_2_, and 10 MgSO_4_. The brain was quickly removed and placed in ice-cold NMDG ACSF. Sagittal slices (320–270 μm) were cut immediately at an angle of 17° to the midline, in order to preserve axonal connections from the STR to the SNr, on a HM 650 V Slicer (MICROM International) and transferred to the holding chamber filled with HEPES holding ACSF for the remainder of the day at room temperature (22–26°C). The HEPES ACSF contained the following (in mM): 92 NaCl, 2.5 KCl, 1.25 NaH_2_PO_4_, 30 NaHCO_3_, 20 HEPES, 25 glucose, 2 thiourea, 5 Na-ascorbate, 3 Na-pyruvate, 2 CaCl_2_, and 2 MgSO_4_, pH 7.4 with 95% O_2_/5%CO_2_.

### *In vitro* Electrophysiology

We performed all recordings with the whole-cell patch-clamp technique on SNr neurons at room temperature. We constantly perfused the recording chamber with oxygenated ACSF containing (in mM): 125 NaCl, 2.5 KCl, 1.25 NaH_2_PO_4_, 15 NaHCO_3_, 25 glucose, 2 CaCl_2_, 1 MgCl_2_, and 0.5 Na-ascorbate, pH 7.4 with 95% O_2_/5%CO_2_. We fabricated patch pipettes (4–8 MΩ) from thick-walled borosilicate glass capillaries (2.0 mm outer diameter, 0.5 mm wall thickness, Hilgenberg, Malsfeld, Germany). The pipette solution contained (in mM): 140 K-gluconate, 10 NaCl, 10 HEPES, 4 MgATP, 0.05 spermin, 5 l-glutathione, 0.2 EGTA, and 0.4 GTP (Sigma, pH 7.2 with KOH). The reference electrode was an Ag–AgCl pellet placed in the bath. Voltage and current signals were amplified by an Axopatch-200B amplifier (Axon Instruments), filtered at 5 kHz and sampled at 20 kHz. In voltage-clamp experiments, we coated pipettes with Sylgard (DOW Corning). We applied electrical stimulation via a monopolar 2–3 KΩ Narylene-coated stainless-steel microelectrode positioned in the GP. The anode was an Ag–AgCl pellet placed in the bath. Stimulation pulses were biphasic 50–1,600 μA currents. Stimulus trains consisted of 10 pulses delivered to the GP at 10–20 Hz. In all the experiments, the ACSF solution contained APV (50 μM) and CNQX (15 μM) to block NMDA and AMPA receptors, respectively. In several experiments, the following drugs were added to the ACSF: GABAzine (20 μM) and tetrodotoxin (TTX, 0.5 μM).

### Data and Statistical Analysis

All off-line analyses were carried out using Matlab R2013a (Mathworks, RRID: SCR_001622) and IgorPro 6.0 (WaveMetrics, RRID: SCR_000325) on a personal computer. All results for each experiment were pooled and displayed as the mean ± SEM unless mentioned otherwise. We calculated spontaneous firing rates and coefficients of variation (CV) from stable recordings lasting 10 s and recorded evoked IPSPs and miniature IPSCs (mIPSC) at a holding potential of −60 mV. We calculated evoked IPSP latency as the time from the end of the stimulation to the onset of the current deflection. We detected mIPSCs with an Offline Sorter program (version 2.8.8; Plexon). We analyzed confocal images with ImageJ (version 1.52a) in conjugation with the Synapse Counter plug-in to identify GABAergic puncta. In all figures comparing between naïve and dopamine depleted animals, we used the Mann–Whitney *U*-test (in all figures *p*-values are denoted as ^*^p < 0.05 and ^**^p < 0.01). The curves in **Figures 3D, 4B** were compared using bootstrap. The bootstrap method is a resampling technique used to estimate mean or standard deviation on a population by sampling a dataset with replacement (Efron and Tibshirani, 1993). We used it to estimate whether the difference between the curves fitted to the data in these figures belonged to the same (null hypothesis) distribution. The data in **Figure 6** was compared using the permutation test.

### Immunohistochemistry

**Figure 1:**
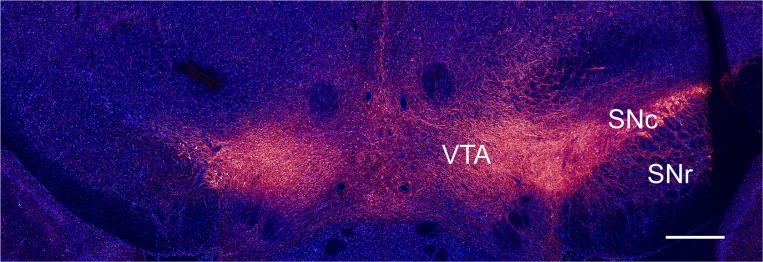
Immunohistochemical verification of unilateral dopamine depletion. Coronal slice from a rat injected with 6-OHDA, immunostained for TH (red) and DAPI (blue), scale bar, 1 mm.

Similar to our previous study (Lavian et al., 2018), we transcardially perfused rats with cold 0.1 M phosphate-buffered saline (PBS) followed by 4% paraformaldehyde (PFA). The brain was removed, post-fixed overnight and then sequentially cryoprotected in 20 and 30% sucrose in 0.1 M PBS until the brain sank in the solution. The fixed brain was sliced into 50 μm- thick coronal slices and sections containing SN were chosen for the next two steps. All immunohistochemistry was completed on free-floating sections and mounted on slides for analysis. In the first step, slices were washed five times in 0.1% Triton X-100 in PBS for a total 25 min. Non-specific binding was blocked with 20% normal goat serum and 0.1% Triton X-100 in PBS for 60 min. Slices were then incubated for 72 h at 4°C with primary antibody diluted in 2% normal goat serum and 0.1% Triton X-100 in PBS. The primary antibodies used were chicken anti-tyrosine hydroxylase for labeling dopaminergic neurons (1:1000, Abcam, Cat# ab76442, RRID: AB_1524535), chicken anti-MAP2 for neuronal labeling (1:1000, Abcam, Cat# ab5392, RRID: AB_2138153), and mouse anti-vGAT for labeling GABAergic presynaptic terminals (1:200, Synaptic Systems, Cat# 131 011, RRID: AB_887872). After incubation with the primary antibody, slices were washed five times in 0.1% Triton X-100 in PBS for a total of 25 min. We performed secondary antibody staining by incubating slices for 1 h at room temperature with the secondary antibody. The secondary antibodies were goat anti-chicken igG conjugated to Alexa 647 for TH and MAP2 staining (1:1000, Abcam, Cat# ab150175, RRID: N/A) and goat anti-mouse conjugated to Alexa 488 for vGAT staining (1:1000, Thermo Fisher Scientific, Cat# A-11001, RRID: AB_2534069). We incubated the slices with Hoechst 33342 (1:1000, Invitrogen) to label nuclei for 10 min and then washed three times in 0.1% Triton X-100 in PBS for a total of 15 min. We then placed the slices on glass slides and allowed them to dry for 15 min, before immersing in a mounting solution and covering with a coverslip. Images were acquired with a Leica SP8 scanning confocal microscope using a 63×/1.4 N.A. oil objective. To generate the coronal image displayed in Figure 1, ~60 tiles were acquired with a 20×/0.75 NA objective at 1024 × 1024 pixels per tile with a zoom of 1.5 and merged into a single image with the Leica LasX acquisition software.

## Results

We studied intrinsic and synaptic plasticity of GABAergic neurons in naïve and dopamine depleted rats. Dopamine depletion was performed using the well-established 6-OHDA model (Ungerstedt, 1968). The toxin was injected unilaterally adjacent to the medial SNc. Ablation of dopaminergic neurons in the SNc was assessed by a behavioral test and by immunohistochemistry. The coronal slice, shown in Figure 1, was immunostained for TH, marking dopaminergic neurons, and DAPI, marking all nuclei. The slice contained the dopamine populated SNc, SNr, and the ventral tegmental area (VTA) regions (Figure 1 right-hand side). The left-hand side of the image depicts the side treated with 6-OHDA, where only the VTA displays staining for TH.

**Figure 2:**
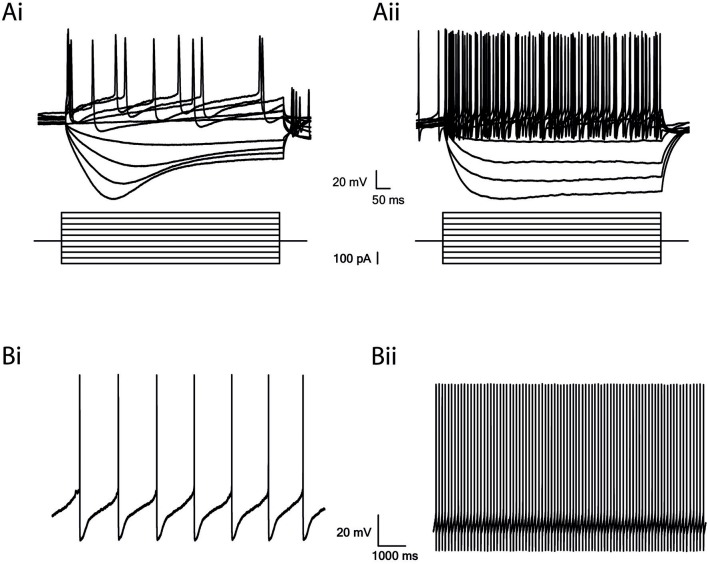
GABAergic and Dopaminergic cells in the SNr have different electrophysiological properties. **(A)** Step recordings of voltage as a response to current injection from −200 to 250 pA (50 pA increments). Low firing rate and a prominent I_h_ current in dopaminergic neurons **(Ai)** and high firing rate and a small or no I_h_ current in GABAergic neurons **(Aii)**. **(B)** Spontaneous activity. Low firing rate of dopaminergic neurons **(Bi)** and high firing rate of GABAergic neurons **(Bii)**.

The SNr contains two major types of neurons: dopaminergic and GABAergic. To investigate the influence of dopamine depletion on GABAergic neurons in the SNr, we used well-established electrophysiological differences to identify the GABA neurons in naïve rats (Hausser et al., 1995). By injecting a negative current, we activated the hyper-polarization-activated current (I_h_) reflected by a sag in the dopamine neurons, in comparison to a small or no sag in the GABA neurons (Figures 2Ai,ii). Also, the firing rate of GABA neurons was faster, and action potentials were shorter than in dopaminergic neurons (Figures 2Bi,ii; Hausser et al., 1995; Seutin and Engel, 2010; Ding et al., 2011). We did not encounter dopaminergic neurons while randomly recording in the SNr from dopamine depleted animals.

**Figure 3:**
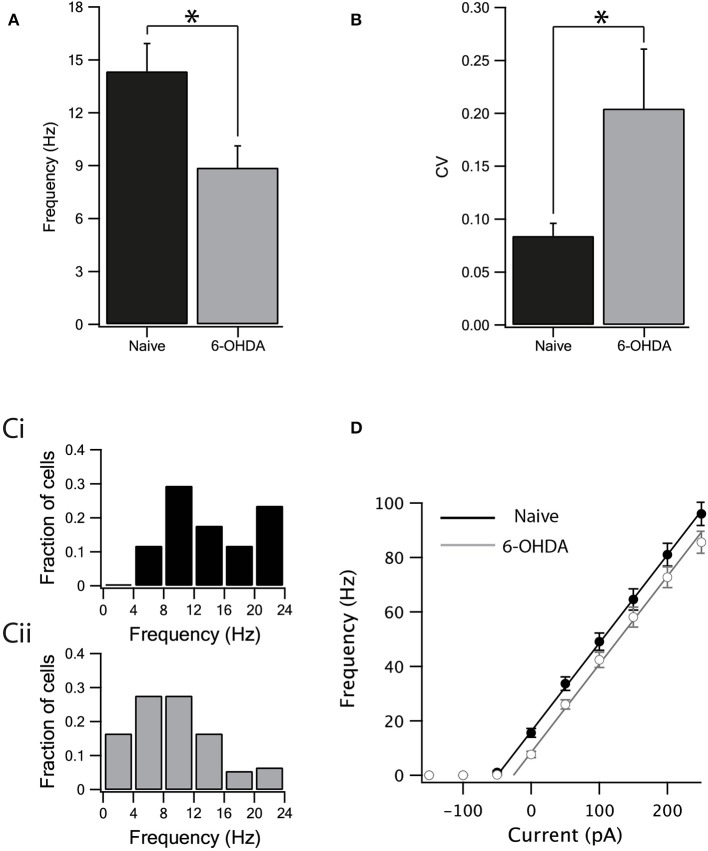
Dopamine depletion decreased the intrinsic excitability of GABA neurons. **(A)** The spontaneous activity of neurons from naïve rats was higher than the activity of neurons from dopamine depleted rats. **(B)** The spontaneous activity of neurons from naïve rats was more regular than the activity of neurons from dopamine depleted rats. **(C)** Spontaneous firing rate distribution recorded from naïve **(Ci)** and parkinsonian rats **(Cii)**. **(D)** Firing frequency as a function of current injection. Straight lines are linear curve fits to the rise phase of the curves. Values are expressed as the mean ± SEM. *p < 0.05, Mann–Whitney *U*-test.

We investigated the effect of dopamine depletion on intrinsic properties of inhibitory neurons after bath application of GABA and glutamate antagonists to block all synaptic transmission. The spontaneous firing rate of GABAergic neurons in the SNr of naïve rats was higher (Figure 3A, naïve: 14.35 ± 1.57 Hz, *n* = 17, 6-OHDA: 8.88 ± 1.23 Hz, *n* = 20, *p* < 0.05, unpaired Mann–Whitney *U*-test) and less regular than in the dopamine depleted rats (Figure 3B, naïve: CV = 0.08 ± 0.01, *n* = 17, 6-OHDA: CV = 0.2 ± 0.05, *n* = 20, *p* < 0.05, Mann–Whitney *U*-test). Accordingly, the distribution of the spontaneous firing rate for dopamine depleted rats (Figure 3Cii) shifted to lower frequencies than that recorded from naïve animals (Figure 3Ci). To further test the changes in firing induced by dopamine depletion, we constructed a frequency-current curve (F-I) by injecting several current steps into each neuron. The F-I curve recorded from neurons from dopamine depleted animals appeared to be shifted to higher current values than that recorded from naïve animals (Figure 3D, naïve: *n* = 17, 6-OHDA: *n* = 20). To estimate this shift in the curve we used linear regression. A straight line was fitted to the rising phase (to account for experimental variability, we used the standard deviations of the points in the curve as weights for the fit) of the I-F curve and its intersection with the current axis was extrapolated. The slopes of the two lines were practically identical (3.2 ± 0.2 for both naïve and 6-OHDA treated animals). The F-I curve of naïve animals crossed the current line at −52 ± 3 pA and that of 6-OHDA treated animals at −29 ± 5 pA. The shift of the 6-OHDA curve was thus estimated to be 23 ± 8 pA. We used bootstrap to test the null hypothesis that this shift was not significant. The weighted curve fit was repeated on 2,000 data sets resampled with replacements and the distribution of the shift in the curve was calculated by rejecting the null hypothesis (*p* < 0.01).

**Figure 4:**
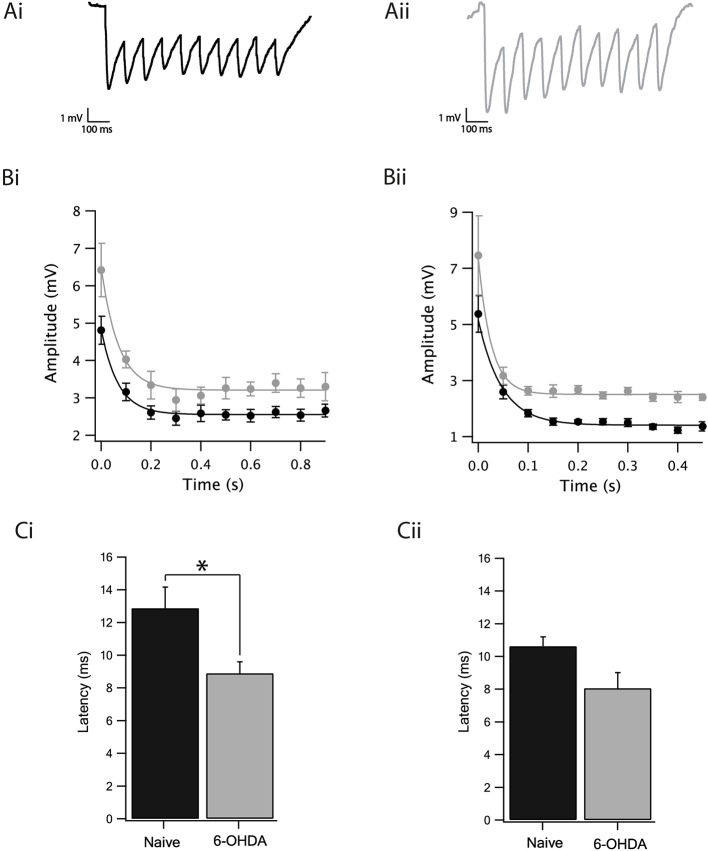
Dopamine depletion increased GABAergic transmission from the GP to the SNr. **(A)** Example of IPSPs recorded after a 10 Hz stimulation train of SNr neurons from naïve **(Ai)** and dopamine depleted rats **(Aii)**. **(B)** Amplitude of the IPSP of naïve (black) dopamine depleted rats (gray) stimulated at 10 Hz **(Bi)** and at 20 Hz **(Bii)**. Lines are exponential curve fits to the data. **(C)** Latency of the IPSP of naïve (black) and dopamine depleted (gray) rats stimulated at 10 Hz **(Ci)** and at 20 Hz **(Cii)**. Values are expressed as the mean ± SEM. *p < 0.05, Mann–Whitney *U*-test.

We observed a slight reduction in the excitability of GABAergic neurons following dopamine depletion when all synaptic transmission to the SNr was blocked. The next step consisted of investigating whether, in addition to the reduction in excitability, there would be a change in inhibitory synaptic transmission to the GP-SNr. We stimulated in the GP by use of an extracellular stimulating electrode and recorded the IPSPs of the SNr GABAergic neurons. In our hands, stimulating the striatum did not generate any synaptic activity in the SNr, probably due to cutting of striatal axons during the slicing procedure. GP-GP, GP-EP, and GP-SNr synapses display short-term depression while that STR-EP and STR-GP synapses displayed short-term facilitation (Connelly et al., 2010; Bugaysen et al., 2013; Lavian and Korngreen, 2016). To investigate the impact of dopamine depletion on connections between the GP and SNr, we retained for analysis only recordings exhibiting short-term depression. Figure 4A illustrates the IPSP recordings from GABAergic neurons as a response to an extracellular stimulus at 10 Hz to brain slices from naïve (Figure 4Ai) and dopamine depleted rats (Figure 4Aii). The amplitude of the steady-state depression of the IPSPs recorded from the dopamine depleted animals, calculated from exponential curve fit to the IPSP trains (using the standard deviation of the points in the curve as weights for the fit and testing for statistical significance as in Figure 3 by bootstrapping the difference in the steady-state depression), was bigger than in naïve ones at both the 10 Hz (Figure 4Bi, 6-OHDA: 3.2 ± 0.1 mV, naïve: 2.5 ± 0.1 mV, *p* < 0.01, bootstrap) and 20 Hz stimulation frequencies (Figure 4Bii, 6-OHDA: 2.5 ± 0.05 mV, naïve: 1.4 ± 0.1 mV, *p* < 0.01, bootstrap). Furthermore, the latency of the first IPSP, calculated as the time between the stimulation and the onset of the IPSP, was shorter in neurons from dopamine depleted animals at 10 Hz (Figure 4Ci, naïve: 12.87 ± 1.7, *n* = 10, 6-OHDA: 8.89 ± 0.7, *n* = 7, *p* < 0.05, Mann–Whitney *U*-test) but not for 20 Hz stimulation (Figure 4Cii, naïve: 10.63 ± 0.58, *n* = 10, 6-OHDA: 8.06 ± 0.95, *n* = 4, *p* = 0.15, Mann–Whitney *U*-test).

**Figure 5:**
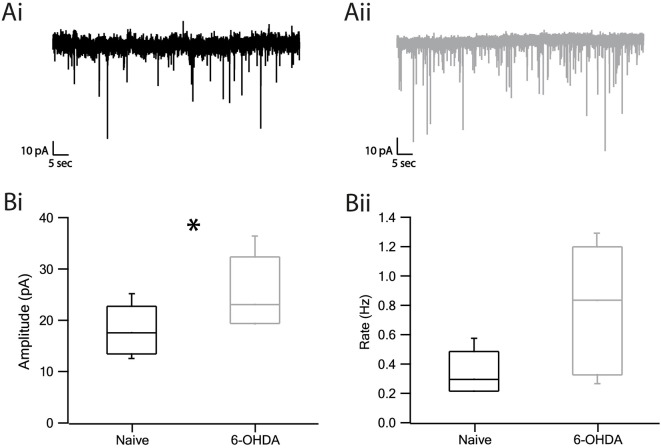
Miniature IPSC amplitude and frequency increased in dopamine depleted rats. **(A)** Representative traces of mIPSC recordings from naïve **(Ai)** and parkinsonian **(Aii)** rats. **(Bi)** Amplitude of mIPSC of naïve and 6-OHDA rats. **(Bii)** Frequency of mIPSC. Values are expressed as the mean. *p < 0.05, Mann–Whitney *U*-test. The central line indicates the median, the box indicates the quartiles and the whiskers are 10 and 90% of the data.

The increase in the amplitude of evoked IPSPs suggested a possible change in the response of SNr neurons to GABA release. To probe this hypothesis, we recorded spontaneous GABAergic release using the voltage-clamp method. Representative examples of the miniature IPSCs are displayed in Figure 5Ai of naïve and Figure 5Aii of dopamine depleted rats. The changes in amplitude and frequency of the mIPSCs (miniature IPSC) in neurons from dopamine depleted rats as compared to naïve rats appear in the boxplot (Figure 5B). The amplitude was found to be larger in lesioned rats than in naïve rats (Figure 5Bi, naïve: 18.07 ± 1.5 pA, *n* = 8, 6-OHDA: 25.3 ± 2.5 pA, *n* = 6, *p* < 0.05, Mann–Whitney *U*-test). The frequency of mIPSCs appeared to be higher in dopamine depleted rats than in naïve subjects but was not statistically significant (Figure 5Bii, naïve: 0.34 ± 0.04 Hz, *n* = 8, 6-OHDA: 0.79 ± 0.15 Hz, *n* = 6, *p* > 0.05, Mann–Whitney *U*-test).

**Figure 6:**
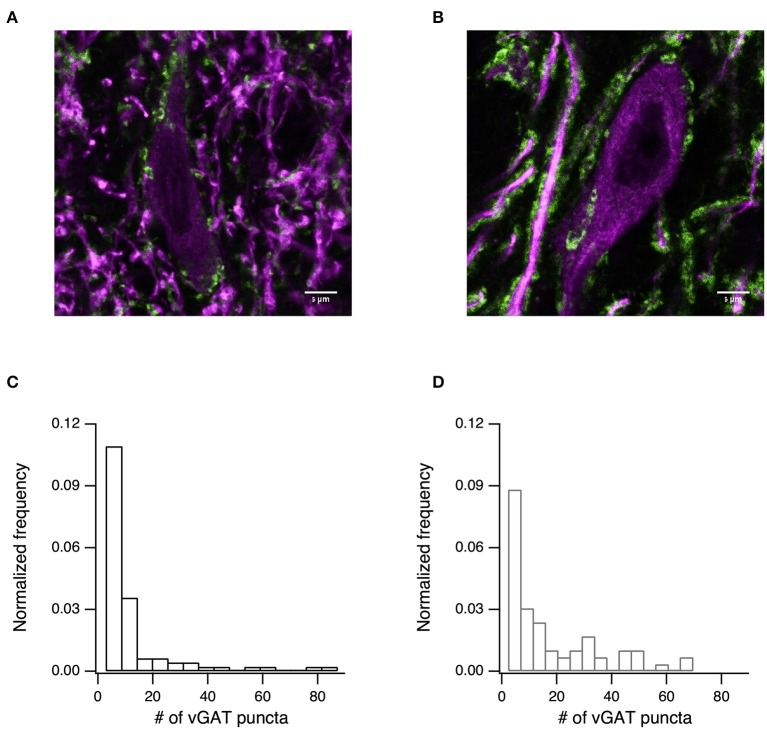
The number of inhibitory terminals increased after dopamine depletion. **(A)** Confocal image showing immunostaining for the inhibitory presynaptic marker vGAT (green), and microtubule marker MAP2 (purple) for naïve **(A)** and dopamine-depleted **(B)** rats. Scale bar is 5 μm. **(C)** Histogram of the distribution of vGAT puncta counted from 84 confocal images taken from 4 naïve rats (mean ± SD: 9.6 ± 15.7 puncta/image, median 4 puncta/image). **(D)** Histogram of the distribution of vGAT puncta counted from 66 confocal images taken from 3 6-OHDA treated rats (mean ± SD: 15.2 ± 17.5 puncta/image, median 8 puncta/image).

Finally, we tested whether the changes to synaptic transmission were due to an increase in the density of the GABAergic synapses. To test this prediction, we immunostained slices for vGAT (Figure 6, green) and MAP2 (Figure 6, purple). Using confocal imaging we took 84 images from the SNr of 4 naïve rats and 66 images from the SNr of 3 6-OHDA treated rats (all images were taken at the same magnification and are of similar areas in the SNr). We then counted the number of inhibitory terminals in the SNr using ImageJ. The number of puncta per image are displayed in Figure 6C for naïve rats (mean ± SD: 9.6 ± 15.7 puncta/image, median 4 puncta/image) and in Figure 6D for 6-OHDA treated rats (mean ± SD: 15.2 ± 17.5 puncta/image, median 8 puncta/image). Permutation tests of the medians rejected the null hypothesis that the medians were from similar distributions (*p* < 0.05, permutation test).

## Discussion

We found that dopamine depletion leads to a decrease in the intrinsic excitability of the GABAergic neurons after blocking synaptic transmission. The activity pattern also changed and displayed a reduction in the regularity of firing. To study the influence of dopamine depletion on inhibitory synaptic transmission, we stimulated the GP and recorded IPSPs in the SNr. We observed an increase in IPSP amplitude and a decrease in its latency in dopamine depleted rats. We examined spontaneous release of GABA to the SNr neurons by recording mIPSCs and found an increase in their amplitude in dopamine depleted as compared to naïve rats. Finally, we counted vGAT stained puncta and observed an increase in their number in dopamine depleted slices. These results may suggest that synaptic proliferation, probably of the GP-SNr pathway but possibly of other pathways, can explain how dopamine depletion augments GABAergic transmission in the SNr.

### Dopamine Depletion Decreases Excitability of GABAergic Neurons in the SNr

Spontaneous firing of GABAergic neurons, in the presence of blockers for synaptic transmission, was affected by dopamine depletion, probably due to changes in intrinsic neuronal properties. The decrease in the firing rate of dopamine depleted rats observed here is consistent with previous work on the SNc-SNr pathway (Zhou et al., 2009) which suggests the existence of a pathway where dopamine released from dendrites of SNc neurons binds to D1 and D5 receptors on GABA neurons in the SNr. The coactivation of the two receptors enhances the activity of TRPC3 channels, thus leading to depolarization. Inhibition of these receptors led to a reduction in firing rate and an increase in the irregularity of ISI, which may imply that in dopamine depleted rats, there was no dendritic release of dopamine, no TRPC3 enhancement and thus a reduction in the spontaneous firing rate in comparison to the naïve rats. These findings are also in line with recent work showing a reduction in firing rate, under two conditions; namely, the blockage of D_1_ and D_2_ receptors and experimental Parkinsonism in juvenile mice (Cáceres-Chávez et al., 2018). We also identified an increase in irregularity, as did Zhou et al. (2009). In contrast to *in vivo* studies that have reported an increase in the bursting activity of SNr neurons (Murer et al., 1997; Lee et al., 2001; Wang et al., 2010b; Cáceres-Chávez et al., 2018), we rarely encountered bursting activity, which may be due to the use of blockers for glutamatergic inputs in our experiments (Ibáñ Ez-Sandoval et al., 2007; Aceves et al., 2011).

### GP-SNr Synaptic Change After Dopamine Depletion

Dopamine has been shown to influence signal transfer between nuclei of the basal ganglia (Miguelez et al., 2012; Dupuis et al., 2013; Lavian et al., 2017). When stimulating the GP nucleus and recording the signal in the SNr, we observed an increase in the IPSP amplitude in dopamine depleted rats. These results are consistent with previous findings showing that adding the D_2_ agonist depressed the GP-SNr synapse (Aceves et al., 2011). The lack of change in PPR may indicate that during a permanent reduction in dopamine, the effect is postsynaptic, in contrast to a short-term absence of dopamine which causes a presynaptic effect. Interestingly, the GP-GP synapses exhibited the same alterations as the GP-SNr synapses after dopamine depletion; i.e., an increase in amplitude without a change in the PPR (Miguelez et al., 2012). As suggested by Miguelez et al. the change in the efficacy of these synapses may be a homeostatic response to the known hyperactivity of the STN in dopamine depleted rats. The latency of the IPSP of GP-SNr synapse was shorter in dopamine depleted rats. Studies have shown that synaptic strength and presynaptic release probability control synaptic latency and that latency is negatively correlated with the amplitude of postsynaptic IPSP (Boudkkazi et al., 2007). These findings strengthen our observation of increased IPSP amplitude after dopamine depletion in the GP-Snr synapse. It would be worthwhile in future work to investigate the STR-SNr synapse in dopamine depleted rats. We hypothesize that following dopamine depletion, the amplitude of the STR-SNr IPSP should decrease as described in Aceves's pharmacological experiments on the STR-SNr synapse and as described in Lavian's study on the synapse of STR and EP, the parallel output nuclei.

### Increased Amplitude and Frequency of mIPSC After Dopamine Depletion

We recorded mIPSCs using the voltage-clamp method. We prevented action potentials with TTX and probably recorded spontaneous release from the STR and GP axons and local axon collaterals from other SNr neurons. We found that the amplitude and the frequency increased in dopamine depleted rats. The increase in the amplitude of mIPSCs as well as induced IPSP (caused by the stimulation of GP) is consistent with the up-regulation of GABA_A_ receptor gene expression in dopamine depleted rats and hence an increase in the number of postsynaptic receptors (Chadha et al., 2000). Other factors, not examined in dopamine depleted rats, may have a direct influence on the mIPSC amplitude, including the volume of packaged GABA in the synaptic vesicles (Frerking et al., 1995) and changes in subunit composition of the receptors which alter the channel properties (Cherubini and Conti, 2001). On the other hand, the increase in the mIPSC frequency may be explained by a presynaptic effect such as a higher density of synaptic terminals.

To better understand the mechanism behind the change in spontaneous release, we tested the density of the vGAT puncta and found increased synaptic density in the treated slices. The GABA synapse may come from the STR or the GP. It would be of interest to explore the density of terminals in the direct and the indirect pathways individually. Overall, the findings contribute to expanding the knowledge of the spectrum of morphological and physiological changes to basal ganglia function induced by dopamine depletion. The changes in the intrinsic and synaptic properties of SNr neurons highlights the complex modifications that occur during experimental Parkinsonism and possibly also in Parkinson's disease.

## Data Availability

The datasets generated for this study are available on request to the corresponding author.

## Ethics Statement

All experimental procedures were approved and supervised by the Bar-Ilan Animal Care and Use Committee and were in accordance with the National Institutes of Health Guide for the Care and Use of Laboratory Animals and the Bar-Ilan University Guidelines for the Use and Care of Laboratory Animals in Research. This study was approved by the Israel National Committee for Experiments in Laboratory Animals at the Ministry of Health. This article does not contain any studies with human participants performed by any of the authors.

## Author Contributions

AF and AJ designed and carried the work and wrote the paper. HL designed and carried the work. AK designed the experiments and wrote the paper.

### Conflict of Interest Statement

The authors declare that the research was conducted in the absence of any commercial or financial relationships that could be construed as a potential conflict of interest.
